# Invasive pneumococcal disease caused by mucoid serotype 3 *Streptococcus pneumoniae*: a case report and literature review

**DOI:** 10.1186/s13104-016-2353-3

**Published:** 2017-01-04

**Authors:** Naomi Sugimoto, Yuka Yamagishi, Jun Hirai, Daisuke Sakanashi, Hiroyuki Suematsu, Naoya Nishiyama, Yusuke Koizumi, Hiroshige Mikamo

**Affiliations:** Department of Clinical Infectious Diseases, Aichi Medical University, 1-1 Yazakokarimata, Nagakute, Aichi 480-1195 Japan

**Keywords:** Invasive Pneumococcal disease, IPD, Mucoid, *Streptococcus pneumoniae*, Serotype 3

## Abstract

**Background:**

Among the different serotypes of *Streptococcus pneumoniae*, serotype 3 has received global attention. We report the fatal case of a 76-year-old Japanese man who had an invasive pneumococcal disease associated with pneumonia caused by serotype 3 *S. pneumoniae*.

**Case presentation:**

The patient had a history of hypertension, laryngeal cancer, chronic obstructive pulmonary disease, and type 2 diabetes mellitus. Following a cerebral arteriovenous malformation hemorrhage, he underwent surgery to remove the hematoma and began rehabilitation. On day 66 of hospitalization, he suddenly developed a fever, and coarse crackles and wheezes were heard in his right lung. A diagnosis of hospital-acquired aspiration pneumonia was made, and initial treatment with piperacillin/tazobactam was started. Teicoplanin was added after *S. pneumoniae* was isolated from the blood culture, however, the patient died 5 days later. The *S. pneumoniae* detected in the sputum smear was serotype 3, showed mucoid colonies and susceptibility to penicillins, cephalosporins, carbapenems, and levofloxacin, but resistance to erythromycin.

**Conclusion:**

We experienced a fatal case of pneumonia caused by mucoid serotype 3 *S. pneumoniae* with a thick capsule. Serotype 3-associated pneumonia may develop a wider pulmonary infiltrative shadow, a prolonged therapeutic or hospitalization course, and a poor outcome. Careful observation and intervention are required, and the use of additional antibiotics or intravenous immunoglobulins should be considered in such cases. Pneumococcal immunization is also an important public health measure to minimize the development of severe infections caused by serotype 3 strains.

## Background

More than 95 different antigenic serotypes of *Streptococcus pneumoniae* are known. Owing to a thicker capsule, greater virulence, and higher mortality rate compared to other strains [1, 2], serotype 3 *S. pneumoniae* has received global attention. We report a case of invasive pneumococcal disease (IPD) associated with pneumonia caused by serotype 3 *S. pneumoniae*, with a dramatic clinical course.

## Case presentation

A 76-year-old Japanese man with a history of hypertension, laryngeal cancer, chronic obstructive pulmonary disease (COPD), and type 2 diabetes mellitus developed a cerebral arteriovenous malformation hemorrhage and was hospitalized at Aichi Medical University Hospital, Japan. His vaccination history was unknown. Case characteristics and laboratory data on the first visit are summarized in Table 1. Following surgery for removal of the hematoma, he began rehabilitation and was encouraged to engage in early postoperative ambulation. In March, 2015, on the 66th day of hospitalization, he developed a sudden fever and exhibited a sharp decline in oxygenation.

**Table 1:** Patient characteristics and the first visit laboratory test findings

*Patient characteristics*	
Height	168.0 cm
Body weight	67.4 kg
Body mass index	23.9 kg/m^2^
*Hematological test*	
White blood cell count	7700/µL
	Neutrophil 86.0%
	Lymphocyte 11.0%
	Monocyte 2.0%
Red blood cell count	441 × 10^4^/µL
Hemoglobin	13.1 g/dL
Platelet count	27.6 × 10^4^/µL
*Blood gas*	
pH	7.182
pCO_2_	53.9 mmHg
pO_2_	118.9 mmHg
HCO_3_^−^	19.5 mmol/L
Lactate	91.6 mg/dL
*Biochemical test*	
Blood urea nitrogen	20.3 mg/dL
Creatinine	1.01 mg/dL
Estimated glomerular filtration rate	55 mL/min/1.73m^2^
Sodium	126 mEq/L
Potassium	4.8 mEq/L
Chloride	90 mEq/L
Total bilirubin	0.14 mg/dL
Aspartate aminotransferase	57 IU/L
Alanine aminotransferase	31 IU/L
Alkaline phosphatase	427 IU/L
Lactate dehydrogenase	402 IU/L
γ-glutamyl transpeptidase	152 IU/L
Cholinesterase	123 IU/L
Creatine phosphokinase	31 IU/L
Albumin	2.5 g/dL
C-reactive protein	16.26 mg/dL
Procalcitonin	13.81 ng/mL

**Fig. 1:**
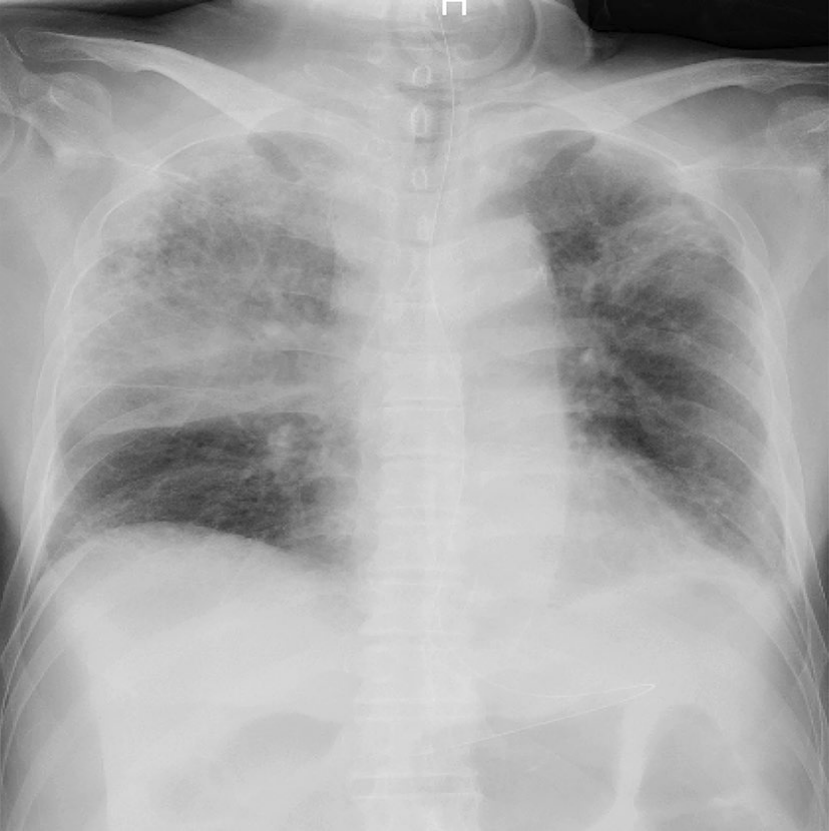
Chest radiography (decubitus) image at the onset of fever

**Fig. 2:**
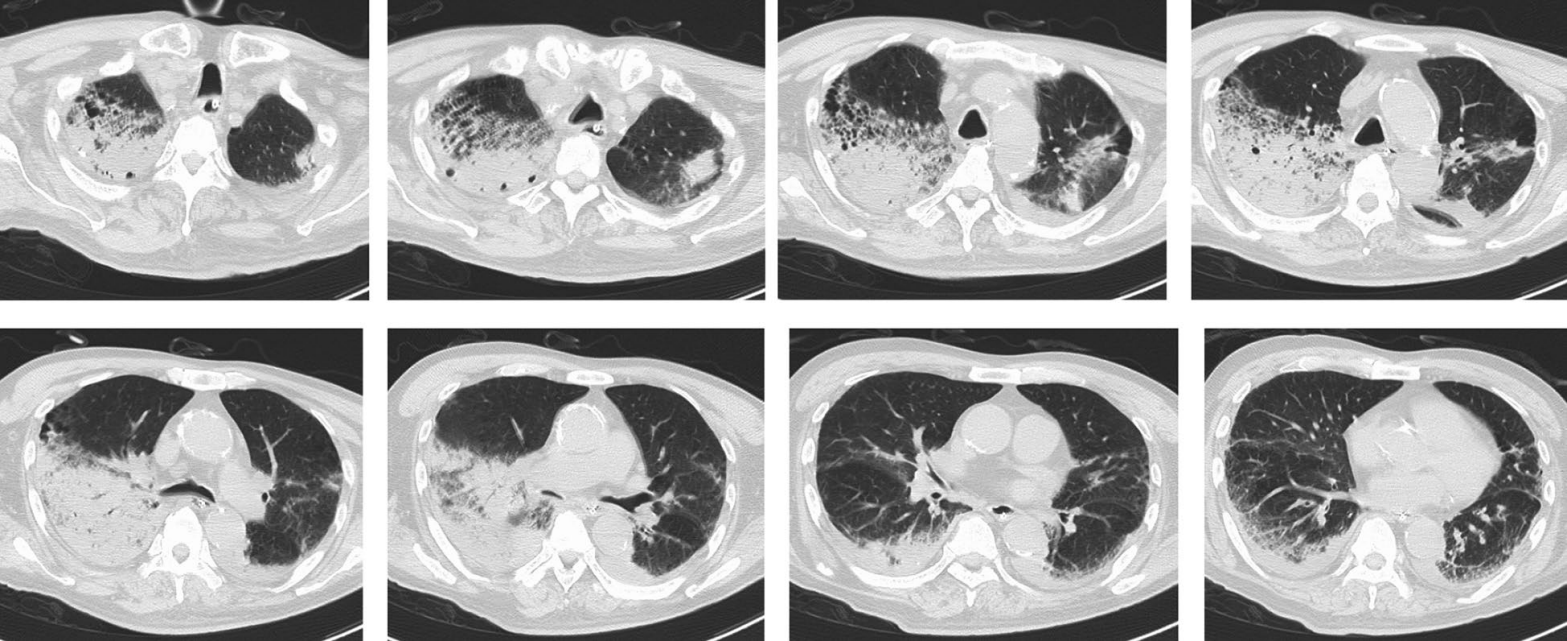
Chest computed tomography images at the onset of fever

At the onset of fever, the patient’s vital signs were as follows: body temperature, 37.8 °C; blood pressure, 84/41 mmHg; heart rate, 107/min; respiration rate, 30/min; and SpO_2_, 82% (room air). Blood gas analysis (room air) showed pH 7.538, ${\text{p}}{{{\text{CO}}_{ 2} }}$ 25.7 mmHg, ${\text{p}}{{{\text{O}}_{ 2} }}$ 47.6 mmHg, HCO_3_^−^ 21.4 mmol/L, and lactate 38.9 mg/dL. His level of consciousness was I-2 on the Japan Coma Scale. Physical examination showed coarse crackles and wheezes in the right lung. Based on chest radiography (Fig. 1) and computed tomography images (Fig. 2), hospital-acquired aspiration pneumonia was diagnosed.

**Fig. 3:**
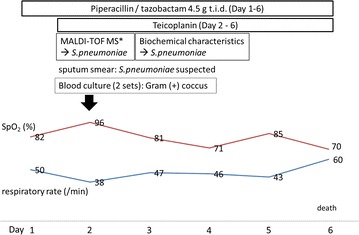
Clinical course of the present case, diagnosed as an invasive pneumococcal disease with pneumonia. *Asterisk* denotes matrix-assisted laser desorption/ionization-time of flight mass spectrometry

Piperacillin/tazobactam 4.5 g was administered three times daily as initial treatment (Fig. 3). *Streptococcus pneumoniae* infection was suspected based on a rapid identification test using a sputum smear, and a strain of *S. pneumoniae* was isolated from the blood culture sampled at the onset of fever. The patient was admitted to the intensive care unit and teicoplanin was added to his treatment regimen. However, his SpO_2_ and respiratory rate continued to be unstable. After 5 days of concomitant teicoplanin administration, the patient died.

**Fig. 4:**
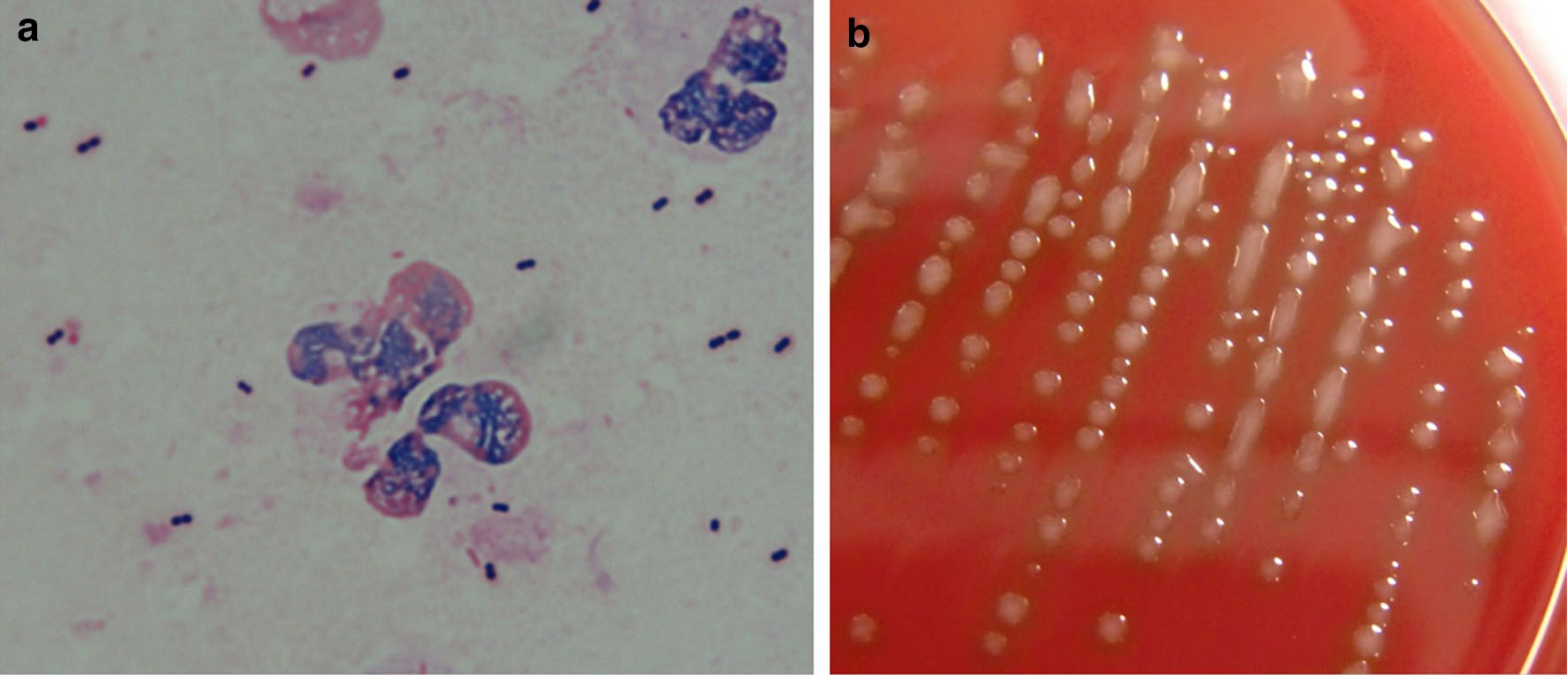
Sputum smear (**a**) and colonies (**b**) of the isolate from the patient showing serotype 3 *Streptococcus pneumoniae.* Capsule is stained *pale pink* (**a**). Mucoid colonies on blood agar are not dimpled (**b**)

*Streptococcus pneumoniae* detected in the smear and the morphologic characteristics of the colonies on blood agar are shown in Fig. 4. The isolate was mucoid serotype strain 3, with a thick capsule. Antibiotic susceptibility to penicillins, cephalosporins, carbapenems, and levofloxacin was good, with resistance observed only to a macrolide (erythromycin) (Table 2).

**Table 2:** Antibiotic susceptibility of the isolated *Streptococcus pneumoniae*

	MIC (µg/mL)^a^	S/I/R^b^
Penicillin G	≤0.063	S
Ampicillin	≤0.25	S
Cefotaxime	≤0.5	S
Cefepime	≤0.5	S
Imipenem	≤0.5	S
Meropenem	≤0.125	S
Levofloxacin	≤1	S
Erythromycin	2	R
Sulfamethoxazole/trimethoprim	≤0.25	S

^a^Minimum inhibitory concentration

^b^Defined as “susceptible”, “intermediate” or “resistant” based on the Clinical And Laboratory Standards Institute standards M100-S25

## Conclusions

*Streptococcus pneumoniae* is encapsulated, which is highly important for its virulence. In particular, serotype 3 strains are reported to be heavily encapsulated compared to other serotypes [1, 2], and tend to form mucoid colonies [3]. These features are related to its high virulence as they protect the bacteria from phagocytosis, inhibit opsonization by complement, and allow it to escape the neutrophil extracellular traps.

Mucoid serotype 3 is the second most common isolate in adult IPD cases. It is reported to be more common in adults with pneumonia, sepsis, and empyema/pleuritis, but not meningitis. In a previous study from Japan, among isolates from 43 adult fatal cases, serotype 3 has remained dominant without significant changes over time [4]. Community-acquired pneumonia caused by mucoid-type pneumococcus is reported to develop a wider infiltrative shadow, higher treatment failure rate, and a longer treatment period or hospitalization than the non-mucoid type [5].

The serotypes of the strains in children and adults are different. An increased prevalence of serotype 3 *S. pneumoniae* among children was reported in one region of Japan after introduction of the 7-valent pneumococcal conjugate vaccine (PCV7) [6]. National surveillance of pediatric patients after the 13-valent pneumococcal conjugate vaccine (PCV13) introduction in Japan showed that the prevalence rates of serotype 3 were 0.8% and 8.5% in IPD and non-IPD patients, respectively, in 2014 [7]. Serotype 3 was not dominant overall, and there was no significant difference in its prevalence rate between 2012 (PCV7 era, 3.7%) and 2014 (PCV13 era, 3.8%).

Serotype 3 has been reported to be dominant among case isolates in adult pneumococcal pneumonia. The Adult Pneumonia Study Group-Japan investigated etiologic factors at four community-based hospitals in four prefectures from September 2011 through January 2013. Of 100 *S. pneumoniae* isolates, serotype 3 was the most dominant (22%), followed by serotypes 11A (10%) and 19F (8%) [8]. In a report on the annual changes in the prevalence of each serotype in lower respiratory samples of adult pneumococcal pneumonia patients from 2011 to 2013, serotype 3 was continuously isolated from 15% or more patients, while the frequency of serotypes 19F, 23F, and 4 decreased annually [9]. Serotype 3 is one of the remaining dominant serotypes in other countries and appears to be more important in older adults on a global level [10]. It should be noted that the prevalence of serotype 3 has not decreased despite higher-valent vaccine introduction.

An outbreak of pneumococcal pneumonia caused by *S. pneumoniae* serotype 3 was reported in a nursing home unit at a local hospital in Kanagawa, Japan, in 2013 [11]. Among 31 residents, 27 (87%) had been vaccinated for influenza in the 2012–13 season, but only 2 (7%) among them had been immunized with the 23-valent polysaccharide pneumococcal vaccine (PPSV23). In total, ten confirmed cases of pneumonia and 16 influenza-like illness (ILI) cases were identified. In the same period, 6 of 28 (attack rate 21%) staff members presented with ILI, but none developed pneumonia. All six *S. pneumoniae* isolates showed identical pulsed-field gel electrophoresis patterns and were susceptible to penicillins, cephalosporins, carbapenems, and vancomycin, and were resistant to erythromycin and clindamycin. All pneumonia patients were hospitalized and none had been vaccinated with PPSV23. Shiramoto et al. reported that the immunogenicity of PCV13 and PPSV23, measured as opsonophagocytic activity titer, for serotype 3 were both lower than that for the other serotypes, suggesting lower vaccination efficacy [12]. Another study reported that the effectiveness of the PCV13 vaccine for serotype 3 was not significant [13]. Although we cannot conclude that the relatively poor efficacy of vaccination is the only reason for the dominance of serotype 3 after the introduction of the higher-valent vaccine, we should take into account the possible variability of immunogenicity depending on the serotype.

The Advisory Committee on Immunization Practices (ACIP) recommended in 2014 that all adults ≥65 years of age should receive PCV13, followed by PPSV23 [14, 15]. This 2-step vaccination approach is intended to maximize the efficacy of pneumococcal vaccination. Initial PCV13 induces acquired T cell memory function, and wider serotype coverage is induced by the subsequent PPSV23 [16]. In Japan, both PPSV23 and PCV13 have been used in the elderly to prevent pneumococcal infections since the approval of extended use of PCV13 in June 2014. The national immunization program launched in October 2014 for those aged ≥65 years only subsidized PPSV23 [17]. Widespread adoption of ACIP recommendations would potentially improve the efficacy of pneumococcal immunization.

Addressing the antibacterial susceptibility, Okade et al. reported that 100% of the 42 serotype 3 strains in their study cohort had penicillin binding protein gene (*pbp*) mutations and macrolide resistance genes [6]. Minimum inhibitory concentrations (MICs) of penicillins are still usually low, even with the *pbp* mutations. The *S. pneumoniae* isolate in the present case was susceptible to penicillins, but resistant to a macrolide (erythromycin). However, the in vivo–in vitro paradox of macrolides has recently been reported. For example, many azithromycin-resistant pneumococcal pneumonia cases have successfully been treated using azithromycin alone [18]. A case of severe community-acquired pneumonia due to mucoid *S. pneumoniae*, that was macrolide-resistant and penicillin-susceptible, was also cured by additional azithromycin administration [16]. Based on the antibiotic susceptibility pattern of *S. pneumoniae*, penicillins are the first treatment choices for infections caused by serotype 3 strains. Macrolides can be considered for concomitant administration in cases in where penicillins do not show sufficient efficacy.

Athlin et al. investigated the relationship between *S. pneumoniae* serotype and immunoglobulin (Ig) titer in community-acquired pneumococcal pneumonia patients [19]. Higher Ig titer ratios were observed in patients infected with serotypes with a thin capsule and medium/high invasive potential (including 1, 7F, 4, 9 N, 9 V, and 14) than in patients infected with serotypes with a thick capsule and low invasive potential (including 3, 6B, 19A, 19F, and 23F). Low Ig titer ratios (<1) were predominantly found in patients infected with serotypes with a thick capsule. According to a report by Hennezel et al. combination therapy with intravenous immunoglobulin (IVIG) and ampicillin was effective in a mouse model of invasive pneumonia caused by serotype 3 *S. pneumoniae* [20]. Based on these results, adding IVIG to the antibiotic therapy regimen may be an option in severe cases of IPD, such as the present case.

As the patient’s immunization status was unclear in the present case, the effectiveness of PPSV23 or PCV13 against serotype 3 *S. pneumoniae* could not be evaluated. The case was further complicated by a history of hypertension, laryngeal cancer, COPD, type 2 diabetes mellitus, and cerebral arteriovenous malformation hemorrhage. In addition, the patient’s nutritional status was not good, as indicated by the low serum albumin level. These factors possibly influenced the disease outcome. However, in severe cases caused by serotype 3 *S. pneumoniae* with a poor treatment response, as the present case, any available treatment measures such as the concomitant use of antibiotics and adding IVIG to the regimen should be considered. Raising awareness and promoting effective pneumococcal immunization would be other potential strategies to minimize the risk of serotype 3 infections.

We experienced a case of nosocomial pneumonia caused by mucoid serotype 3 *S. pneumoniae* with a fulminant course. The isolate was susceptible to all tested antibiotics other than erythromycin, but the case was not managed successfully. Serotype 3 isolates tend to have a thick capsule, and infected patients often have severe and refractory IPD. In particular, pneumonia due to serotype 3, which tends to form mucoid colonies, may develop a wider pulmonary infiltrative shadow, a prolonged therapeutic or hospitalization period, and a poor outcome. Careful observation and intervention are required throughout the clinical course in such patients. Although the complicated background influenced the current patient’s outcome, additional use of other antibiotics or immunoglobulins could be considered as therapeutic options when the pathogen is serotype 3 or another mucoid pneumococcus. In addition, effective and appropriate immunization for *S. pneumoniae* is an important public health measure to minimize the development of severe infections caused by serotype 3 strains.

## Abbreviations

IPD: invasive pneumococcal disease
COPD: chronic obstructive pulmonary disease
PCV7: 7-valent pneumococcal conjugate vaccine
PCV13: 13-valent pneumococcal conjugate vaccine
PPSV23: 23-valent polysaccharide pneumococcal vaccine
ILI: influenza-like illness
ACIP: Advisory Committee on Immunization Practices
*pbp* (gene): penicillin binding protein
MIC: minimum inhibitory concentration
IVIG: intravenous immunoglobulin
